# Early Enhanced Leucine-Rich *α*-2-Glycoprotein-1 Expression in Glomerular Endothelial Cells of Type 2 Diabetic Nephropathy Model Mice

**DOI:** 10.1155/2018/2817045

**Published:** 2018-11-01

**Authors:** Sona haku, Hiromichi Wakui, Kengo Azushima, Kotaro Haruhara, Sho Kinguchi, Kohji Ohki, Kazushi Uneda, Ryu Kobayashi, Miyuki Matsuda, Takahiro Yamaji, Takayuki Yamada, Shintaro Minegishi, Tomoaki Ishigami, Akio Yamashita, Kenichi Ohashi, Kouichi Tamura

**Affiliations:** ^1^Department of Medical Science and Cardiorenal Medicine, Yokohama City University Graduate School of Medicine, Yokohama, Japan; ^2^Cardiovascular and Metabolic Disorders Program, Duke-NUS Medical School, Singapore; ^3^Division of Nephrology and Hypertension, Department of Internal Medicine, The Jikei University School of Medicine, Tokyo, Japan; ^4^Department of Molecular Biology, Yokohama City University Graduate School of Medicine, Yokohama, Japan; ^5^Department of Molecular Pathology, Yokohama City University Graduate School of Medicine, Yokohama, Japan

## Abstract

Abnormal angiogenesis plays a major role in the development of early stage diabetic nephropathy. Vascular endothelial growth factor (VEGF) is a classical proangiogenic factor that regulates abnormal glomerular angiogenesis linked to glomerular hypertrophy in the early stage of diabetic nephropathy. Leucine-rich *α*-2-glycoprotein-1 (LRG1) was recently reported as a novel proangiogenic factor that is expressed in endothelial cells and promotes angiogenesis by modulating the transforming growth factor-*β* signaling pathway. However, the pathophysiology of LRG1 in diabetic nephropathy remains largely unknown. In the present study, we investigated intrarenal expression of the novel proangiogenic factor LRG1 in diabetic* db/db* mice by immunohistochemistry and a laser capture microdissection method during the development of diabetic nephropathy. We hypothesized that glomerular LRG1 expression is increased earlier than VEGF expression under conditions of pathological angiogenesis in the early stage of diabetic nephropathy. Thus, we compared glomerular expression of VEGF and LRG1 in diabetic* db/db* mice at 16 and 24 weeks of age. At 16 weeks, diabetic* db/db* mice exhibited glomerular hypertrophy with abnormal angiogenesis characterized by endothelial cell proliferation, which was concomitant with an increase in LRG1 expression of glomerular endothelial cells. However, glomerular VEGF expression was not increased at this early stage. At 24 weeks, the features of early diabetic nephropathy in* db/db* mice had developed further, along with further enhanced glomerular LRG1 expression. At this late stage, glomerular VEGF and fibrosis-related-gene expression was also significantly increased compared with nondiabetic* db/m* mice. These results suggest that LRG1 plays a pivotal role in the initial development of diabetic nephropathy by promoting abnormal angiogenesis, thereby suggesting that LRG1 is a potential preemptive therapeutic target of diabetic nephropathy.

## 1. Introduction

Diabetic nephropathy has become a leading cause of end stage renal disease (ESRD) worldwide [1, 2]. Despite treatment by inhibition of the renin angiotensin system and tight glycemic control, the risk of ESRD remains high. Diabetic nephropathy is diagnosed at the early stage by detection of microalbuminuria. However, early prevention of diabetic nephropathy progression remains challenging. Therefore, understanding the pathogenesis of early stage diabetic nephropathy and developing methods to control its progression are important issues.

Abnormal angiogenesis plays a major role in the development of early stage diabetic nephropathy [3, 4]. It is associated with glomerular hypertrophy and urinary albumin excretion [5–7]. Morphological changes in capillaries, such as elongation and an increased number, contribute to glomerular hypertrophy [3, 4]. In these aberrant vessels, the endothelial cells are swollen and immature, leading to increased vascular permeability [5, 8, 9].

Vascular endothelial growth factor (VEGF) is a critical regulator of abnormal angiogenesis, and its glomerular expression is involved in the pathogenesis of early stage diabetic nephropathy [10–12]. VEGF is mainly expressed in podocytes, and its receptor, vascular endothelial growth factor receptor-2 (VEGFR-2), is expressed in glomerular endothelial cells [13]. VEGF-VEGFR-2 signaling is upregulated in the diabetic glomerulus, which leads to abnormal angiogenesis and endothelial cell proliferation [14].

Very recently, Leucine-rich *α*-2-glycoprotein-1 (LRG1), a novel proangiogenic factor expressed in endothelial cells [15], has been reported to be involved in the development of diabetic nephropathy. Transcriptomic and* in vitro* analyses revealed that LRG1 in glomerular endothelial cells is one of the key regulators in the development of abnormal angiogenesis at the early stage of diabetic nephropathy [16]. However, the pathophysiology of LRG1 in diabetic nephropathy is still largely unknown.

The aim of this study was to compare glomerular expression of the classical proangiogenic factor VEGF and novel proangiogenic factor LRG1 in the early stage of diabetic nephropathy. We investigated glomerular expression of VEGF and LRG1 in a mouse model of diabetes (C57BL/KsJ-db/dbJcl;* db/db* mouse) and compared the changes in expression at 16 and 24 weeks of age to evaluate their association with diabetic nephropathy development.

## 2. Materials and Methods

### 2.1. Animals

This study was performed in accordance with the National Institutes of Health guidelines for the use of experimental animals. All animal experiments were reviewed and approved by the Animal Studies Committee of Yokohama City University. Male* db/db* mice and their age-matched nondiabetic* db/m* (C57BL/KsJ) littermates were purchased from CLEA Japan. The mice were housed in a controlled environment with a 12-hour light-dark cycle at 25°C. The mice were allowed free access to food and water. Mice were fed a standard diet (0.3% NaCl, 3.6 kcal/g, and 13.3% energy as fat; Oriental MF, Oriental Yeast Co, Ltd.). The mice were sacrificed at 16 and 24 weeks of age.

### 2.2. Biochemical Assays

Blood glucose was measured in blood obtained by tail vein puncture. Blood samples were also collected by cardiac puncture when mice were sacrificed in a fed state as described previously [17, 18]. Whole blood was centrifuged at 800 ×* g* for 10 minutes at 4°C to separate the plasma. The resulting plasma was stored at -80°C until use. Plasma creatinine and urinary creatinine were measured using an autoanalyzer (Hitachi 7180; Hitachi, Tokyo, Japan). Urinary albumin was measured by an ELISA kit (Fujifilm Wako Shibayagi, Gunma, Japan).

### 2.3. Metabolic Cage Analysis

To collect urine, metabolic cage analysis was performed as described previously [19, 20]. Mice were provided with free access to tap water and fed a standard diet.

### 2.4. Histological and Immunohistochemical Analyses

Kidneys were fixed with 4% paraformaldehyde and embedded in paraffin. Sections of 4 *μ*m in thickness were stained with periodic acid Schiff (PAS). Immunohistochemistry was performed as described previously [17, 18, 21]. Briefly, the paraffin-embedded sections were dewaxed and rehydrated. Antigen retrieval was performed by microwave heating. The sections were treated for 60 minutes with 10% normal goat serum in phosphate-buffered saline and blocked for endogenous biotin activity using an AVIDIN/BIOTIN Blocking kit (Vector Laboratories, CA, USA). The sections were then incubated with one of the following antibodies: (1) anti-LRG1 antibody (TAKARA, Japan) diluted at 1:100 or (2) anti-CD34 antibody (BD Pharmingen, Japan) diluted at 1:100. The sections were incubated for 60 minutes with biotinylated goat anti-rabbit IgG (Nichirei Corporation, Tokyo, Japan), blocked for endogenous peroxidase activity by incubation with 0.3% H_2_O_2_ for 20 minutes, treated for 30 minutes with streptavidin and biotinylated peroxidase (DAKO, Heidelberg, Germany), and then exposed to hematoxylin, dehydrated, and mounted. To evaluate the glomerular area, 25 glomeruli per mice were measured and averaged. All images were acquired using a BZ-9000 microscope (Keyence).

### 2.5. Real-Time Quantitative PCR Analysis

Total RNA was extracted from the kidney with ISOGEN (Nippon Gene, Tokyo, Japan), and cDNA was synthesized using the SuperScript III First-Strand System (Invitrogen, Carlsbad, CA, USA). Real-time quantitative PCR (RT-qPCR) was performed by incubating the reverse transcription product with TaqMan Universal PCR Master Mix and TaqMan probes (Applied Biosystems, Foster City, CA, USA), as described previously [22, 23]. mRNA levels were normalized to 18S rRNA as a control.

### 2.6. Laser Capture Microdissection and Subsequent RT-qPCR Analysis

Laser capture microdissection (LMD) was performed using a Leica LMD System (LMD 6000), as described previously [24, 25]. Briefly, formalin-fixed, paraffin-embedded tissues were cut into 10 *μ*m-thick sections, mounted on polyethylene terephthalate membrane slides, and stained with hematoxylin-eosin. Next, renal glomeruli were microdissected using the LMD 6000 laser microdissection microscope. In total, 350 glomeruli were microdissected from the renal cortex per mouse. Total RNA was extracted from microdissected tissue using the RNeasy FFPE Kit (Qiagen, Hilden, Germany). cDNA was synthesized using the SuperScript III First-Strand System and applied to TaqMan RT-qPCR analysis.

### 2.7. Statistical Analysis

Statistical analysis was performed using GraphPad Prism software (GraphPad Software, La Jolla, CA, USA). All quantitative data are expressed as the mean ± SEM. Differences were analyzed using the unpaired Student's* t*-test. Values of* P* < 0.05 were considered as statistically significant.

## 3. Results

### 3.1. Characteristics of 16- and 24-Week-Old db/db Mice

Body weight, blood glucose, kidney weight, and the albuminuria level were significantly higher in* db/db* mice compared with* db/m* mice at 16 weeks of age (Table 1). At 24 weeks of age, body weight, blood glucose, kidney weight, and the albuminuria level were significantly increased in* db/db* mice compared with* db/m* mice. However, serum creatinine levels were identical in* db/db* and* db/m* mice at both 16 and 24 weeks of age (Table 1).

### 3.2. Glomerular Hypertrophy with Endothelial Cell Proliferation in db/db Mice

**Figure 1:**
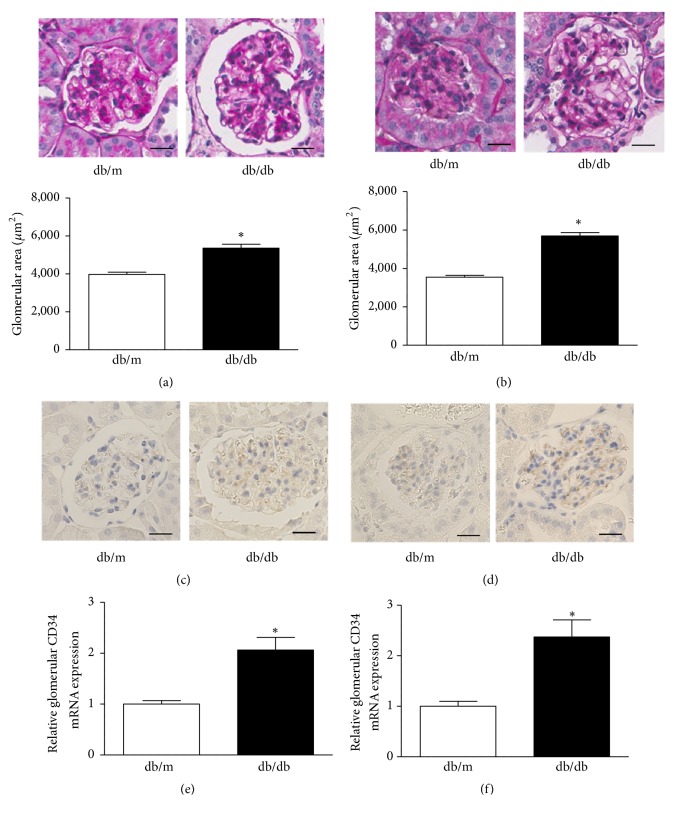
**Glomerular hypertrophy with endothelial cell proliferation in* db/db* mice.** Representative image of PAS staining in glomeruli of* db/m* and* db/db* mice at (a) 16 weeks and (b) 24 weeks of age. The glomerular area was increased in* db/db* mice at both ages (25 glomeruli per mice were measured and averaged). Values are expressed as the mean ± SEM (n = 4–6 in each group). *∗P* < 0.05 versus* db/m*, unpaired* t-*test. Original magnification: ×200, bars = 20 *μ*m. Representative image of CD34 immunostaining at (c) 16 weeks and (d) 24 weeks of age. Quantitative analysis of CD34 in 350 glomeruli identified by an LMD method at (e) 16 weeks and (f) 24 weeks of age. Values are expressed as the mean ± SEM (n = 4–6 in each group). *∗P < *0.05 versus* db/m*, unpaired* t-*test.

At 16 weeks of age,* db/db* mice exhibited a significantly larger glomerular area compared with* db/m* mice (3974.0 ± 117.6 *μ*m^2^ versus 5359.0 ± 202.3 *μ*m^2^,* P* < 0.001) (Figure 1(a)). At 24 weeks of age, the glomerular area had increased further in* db/db* mice compared with* db/m* mice (3542.2 ± 95.2 *μ*m^2^ versus 5689.2 ± 182.5 *μ*m^2^,* P < *0.0001) (Figure 1(b)). We next examined CD34 immunostaining, as an endothelial cell marker, in the glomeruli of* db/db* and* db/m* mice. The immunostaining analysis showed enhancement of glomerular CD34 expression in* db/db* mice compared with* db/m* mice at 16 weeks of age (Figure 1(c)), and this trend became robust at 24 weeks of age (Figure 1(d)). These increases in glomerular endothelial cells indicated that abnormal angiogenesis had occurred in the kidneys of diabetic* db/db* mice [3, 26]. To confirm the increase of glomerular CD34 expression in* db/db* mice, we next examined CD34 mRNA expression in glomeruli of* db/db* and* db/m* mice using an LMD method. Glomerular CD34 mRNA expression was significantly increased in* db/db* mice compared with* db/m* mice at 16 and 24 weeks of age (Figures 1(e) and 1(f)). These results indicate that diabetic* db/db* mice exhibit glomerular hypertrophy with endothelial cell proliferation from the early stage of their lives, and these features are exacerbated along with aging.

### 3.3. Enhanced LRG1 Immunostaining in Glomerular Endothelial Cells of db/db Mice at 16 Weeks of Age

**Figure 2:**
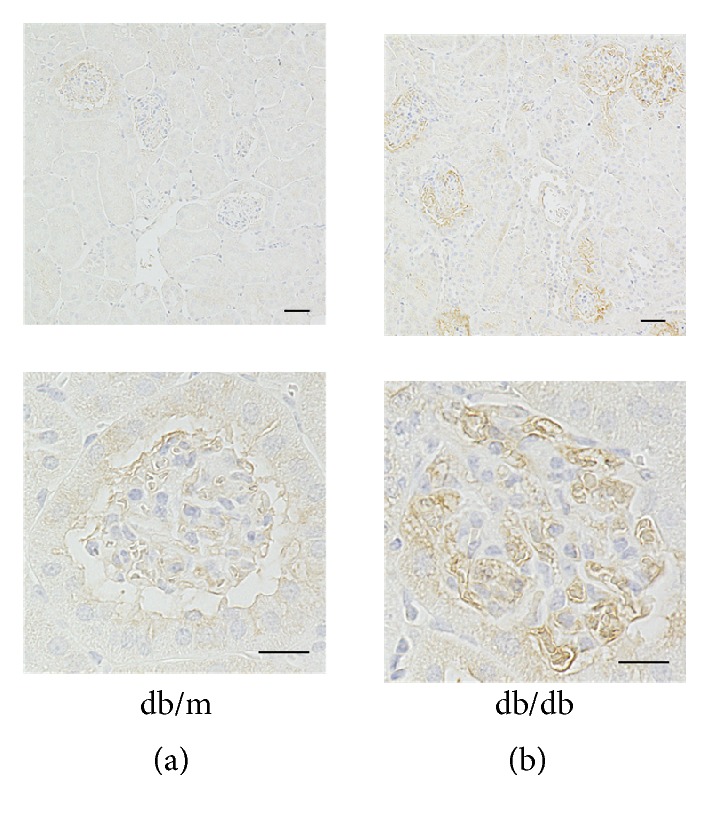
**Enhanced LRG1 immunostaining in glomerular endothelial cells of* db/db* mice at 16 weeks of age.** Representative image of LRG1 immunostaining at 16 weeks of age in (a)* db/m* and (b)* db/db* mice. Lower panels are higher magnifications of immunohistochemical staining for LRG1 in glomerular endothelial cells. Upper panels: original magnification: ×40, bars = 20 *μ*m. Lower panels: original magnification: ×400, bars = 40 *μ*m.

We next examined LRG1 expression and distribution in the renal cortex of* db/db* and* db/m* mice at 16 weeks of age by immunohistochemical analysis. The results showed that* db/db* mice exhibited an increase in LRG1 expression of glomerular endothelial cells, whereas LRG1 was weakly expressed in glomerular endothelial cells of* db/m* mice (Figures 2(a) and 2(b)). LRG1 was also weakly expressed in tubular epithelial cells of renal tubules in* db/db* and* db/m* mice. However, LRG1 expression was not found in podocytes of* db/db* and* db/m* mice. These results indicate that an increase in LRG1 expression of glomerular endothelial cells might be associated with the development of diabetic nephropathy in* db/db* mice.

### 3.4. Enhanced Glomerular LRG1 mRNA Expression in db/db Mice at 16 Weeks of Age

**Figure 3:**
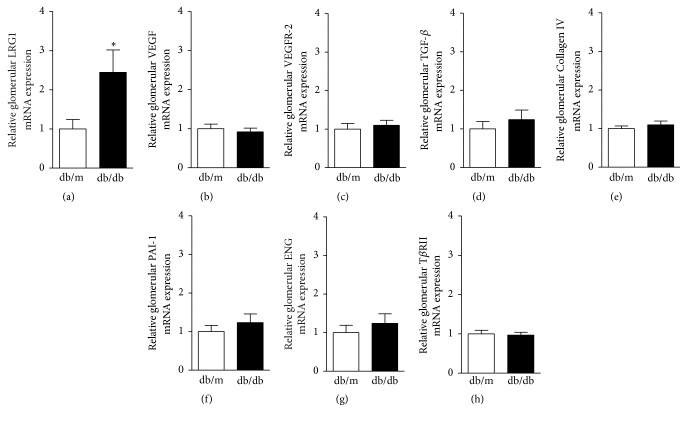
**Enhanced glomerular LRG1 mRNA expression in* db/db* mice at 16 weeks of age.** Quantitative analysis of angiogenesis- and fibrosis-related gene expression in glomeruli identified by the LMD method in* db/m* and* db/db* mice at 16 weeks of age. (a) LRG1, (b) VEGF, (c) VEGFR-2, (d) TFG-*β*, (e) Collagen IV, (f) PAI-1, (g) ENG, and (h) T*β*RII. Values are expressed as the mean ± SEM (n = 4–6 in each group). *∗P* < 0.05, versus* db/m* mice, unpaired* t*-test.

We next examined gene expression in glomeruli of* db/db* and* db/m* mice at 16 weeks of age using the LMD method. Glomerular LRG1 mRNA expression was significantly increased by approximately 2.5-fold in* db/db* mice compared with* db/m* mice (1.0 ± 0.24 versus 2.4 ± 0.57,* P < *0.05) (Figure 3(a)). Intriguingly, glomerular mRNA expression levels of VEGF and its main receptor, VEGFR-2, which are key mediators of abnormal angiogenesis and glomerular hypertrophy, were not increased in* db/db* mice compared with* db/m* mice (Figures 3(b) and 3(c)). Furthermore, glomerular mRNA expression levels of fibrosis-related genes, such as transforming growth factor-*β* (TGF-*β*), Collagen type IV (Collagen IV), and plasminogen activator inhibitor-1 (PAI-1), were identical in* db/db* and* db/m* mice (Figures 3(d)–3(f)). Since LRG1 directly binds to the TGF-*β* accessory receptor endoglin (ENG), and then the complex binds to TGF-*β* receptor-II (T*β*RII), we also examined glomerular mRNA expression of ENG and T*β*RII in* db/db* and* db/m* mice. However, there was no significant difference in glomerular mRNA expression levels of ENG and T*β*RII between* db/db* and* db/m* mice (Figures 3(g) and 3(h)). These results showed that the increase in LRG1 expression preceded the increase in expression of other angiogenesis- and fibrosis-related genes in the glomerulus of diabetic* db/db* mice.

### 3.5. Enhanced LRG1 Immunostaining in Glomerular Endothelial Cells of db/db Mice at 24 Weeks of Age

**Figure 4:**
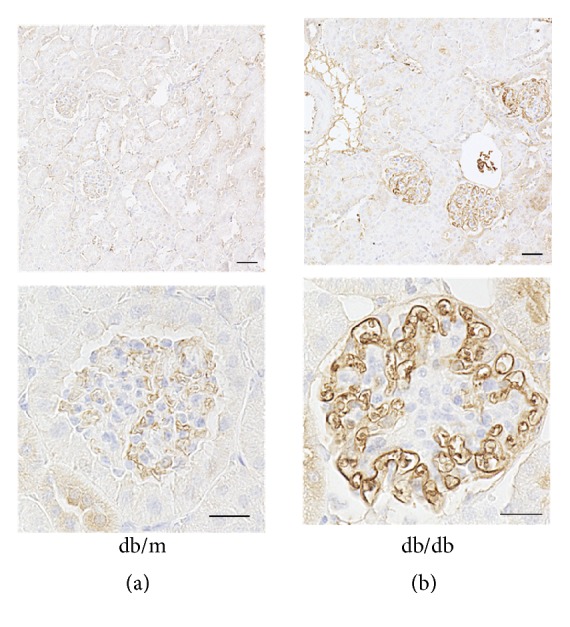
**Enhanced LRG1 immunostaining in glomerular endothelial cells of* db/db* mice at 24 weeks of age.** Representative image of LRG1 immunostaining at 24 weeks of age in (a)* db/m* and (b)* db/db* mice. Lower panels represent higher magnifications of immunohistochemical staining for LRG1 in glomerular endothelial cells. Upper panels: original magnification: ×40, bars = 20 *μ*m. Lower panels: original magnification: ×400, bars = 40 *μ*m.

We next examined LRG1 expression in the renal cortex of* db/db* and* db/m* mice at 24 weeks of age by immunohistochemical analysis. The results showed that LRG1 expression was further enhanced in glomerular endothelial cells of* db/db* mice compared with* db/m* mice at 24 weeks of age (Figures 4(a) and 4(b)).

### 3.6. Increases in Glomerular LRG1, VEGF, and Fibrosis-Related Gene Expression of db/db Mice at 24 Weeks of Age

**Figure 5:**
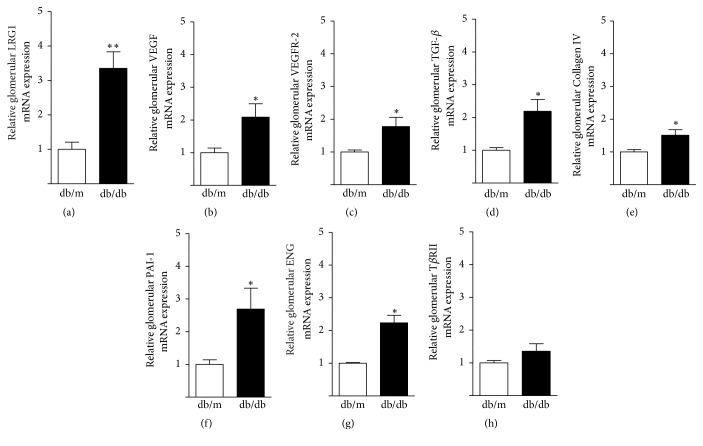
**Enhanced glomerular LRG1, VEGF, and fibrosis-related gene expression in* db/db* mice at 24 weeks of age.** Quantitative analysis of angiogenesis- and fibrosis-related gene expression in glomeruli identified by the LMD method in* db/m* and* db/db* mice at 24 weeks of age. (a) LRG1, (b) VEGF, (c) VEGFR-2, (d) TGF-*β*, (e) Collagen IV, (f) PAI-1, (g) ENG, and (h) T*β*RII. Values are expressed as the mean ± SEM (n = 4–6 in each group). *∗P* < 0.05, *∗∗P* < 0.01 versus* db/m* mice, unpaired* t*-test.

Finally, we examined gene expression in glomeruli of* db/db* and* db/m* mice at 24 weeks of age. Glomerular LRG1 mRNA expression was further increased by approximately 3.5-fold in* db/db* mice compared with* db/m* mice (1.0 ± 0.21 versus 3.40 ± 0.48,* P* < 0.01) (Figure 5(a)). Glomerular VEGF and VEGFR-2 mRNA expression was also significantly increased in* db/db* mice compared with* db/m* mice (Figures 5(b) and 5(c)). In addition, the glomerular expression levels of fibrosis-related genes, such as TGF-*β*, Collagen IV, and PAI-1, were significantly increased in* db/db* mice compared with* db/m* mice (Figures 5(d)–5(f)). Similarly, glomerular mRNA expression levels of ENG and T*β*RII were significantly increased in* db/db* mice compared with* db/m* mice (Figures 5(g) and 5(h)). These results indicated that diabetic nephropathy had developed further after the preceding increase in glomerular LRG1 expression of* db/db* mice.

## 4. Discussion

In the present study, we investigated the intrarenal expression and distribution of a novel proangiogenic factor, LRG1, along with the development of early stage diabetic nephropathy. Our most important finding is that the increase in LRG1 expression of glomerular endothelial cells precedes the increase in VEGF expression, which is another proangiogenic factor involved in the development of diabetic nephropathy. This finding suggests that LRG1 plays a major role in the initial development of diabetic nephropathy by promoting abnormal angiogenesis and glomerular hypertrophy.

In diabetic* db/db* mice at 16 and 24 weeks of age, they exhibited moderate increases in albuminuria levels and glomerular hypertrophy without nodular glomerulosclerosis. These data indicate that our diabetic mice represented the features of early stage diabetic nephropathy, as reported previously [27]. Notably, at 16 weeks of age, glomerular LRG1 expression in diabetic mice was elevated despite glomerular VEGF expression being not increased yet. Considering the results indicating that the glomerular volume and endothelial cells were significantly increased in diabetic mice at this early age, abnormal angiogenesis would already exist in the glomerulus. Thus, there is the possibility that the abnormal angiogenesis observed in the very early stage of diabetic nephropathy in* db/db* mice was attributed to LRG1.

The interaction between LRG1 and VEGF is still unclear, but some studies have shown a possible link between these two factors. In the retina of LRG1 knockout mice, VEGF expression is significantly decreased compared with wild-type mice [15]. In colorectal cancer cells, LRG1 directly induces VEGF expression and promotes tumor angiogenesis [28]. However, LRG1 also exerts proangiogenic effects independently of the VEGF signaling pathway. Double blockade of LRG1 and VEGF suppresses angiogenesis more efficiently than single blockade in the mouse model of choroidal neovascularization [15]. In this study, we demonstrated for the first time that glomerular LRG1 expression was increased earlier than VEGF and VEGFR-2 expression under the conditions of pathological angiogenesis in the early stage of diabetic nephropathy. This finding suggests that glomerular LRG1 is involved in the pathogenesis of abnormal angiogenesis independently of the VEGF signaling pathway, at least in the very early stage of diabetic nephropathy.

Although LRG1 binds directly to ENG and activates the ALK1-Smad1/5/8 pathway for angiogenesis, the TGF-*β*-mediated Smad2/3 pathway is subsequently activated as a counterbalance against angiogenesis to induce quiescence in the endothelium [29, 30]. This vicious pathological cycle of angiogenesis and quiescence finally leads to glomerular fibrosis in diabetic nephropathy [31–34]. In our study, 24-week-old* db/db* mice exhibited upregulation of glomerular fibrosis-related genes, which was concomitant with glomerular hypertrophy and endothelial cell proliferation. It is assumed that the LRG1-induced abnormal angiogenesis observed in 16-week-old* db/db* mice resultantly led to glomerular fibrosis at the later stage.

Our study did not demonstrate whether the increased glomerular LRG1 expression actually caused the abnormal angiogenesis in diabetic* db/db* mice. Therefore, the functional role of LRG1 in abnormal angiogenesis in the early stage of diabetic nephropathy must be investigated further using LRG1 transgenic and knockout mice. Nevertheless, the findings of the present study provide important information regarding the pathogenesis of abnormal angiogenesis in early stage diabetic nephropathy, suggesting that LRG1 is a novel preemptive therapeutic target in diabetic nephropathy.

## 5. Conclusions

Diabetic* db/db* mice exhibited glomerular hypertrophy with abnormal angiogenesis characterized by endothelial cell proliferation at 16 weeks of age, concomitant with an increase in LRG1 expression of glomerular endothelial cells. However, glomerular VEGF expression was not increased at this early stage. The features of early diabetic nephropathy in* db/db* mice had developed further, along with further enhanced glomerular LRG1 expression at 24 weeks of age. Glomerular VEGF and fibrosis-related-gene expression was also significantly increased at this late stage compared with nondiabetic* db/m* mice. These data demonstrate that LRG1 might be pivotal for the initial development of diabetic nephropathy by promoting abnormal angiogenesis.

## Figures and Tables

**Table 1:** Body weight, blood glucose, kidney weight, plasma creatinine, and albuminuria of *db/db* and* db/m mice*.

Variable	16W		24W	
	*db/m*	*db/db*	*db/m*	*db/db*
	(n=6)	(n=6)	(n=4)	(n=4)
Body weight (g)	29.2 ± 1.3	48.2 ± 0.7*∗∗*	37.0 ± 0.5	52.2 ± 4.8*∗*
Blood glucose (mg/dl)	134.8 ± 5.8	>600*∗∗*	130 ± 6.7	574 ± 26*∗∗*
Kidney weight (mg)	182.3 ± 6.7	217.3 ± 8.5*∗∗*	225.0 ± 6.7	263.3 ± 5.8*∗∗*
Plasma creatinine (mg/dl)	0.12 ± 0.22	0.11 ± 0.1	0.08 ± 0.01	0.09 ± 0.02
Albuminuria (mg/mg·Cr)	0.25 ± 0.02	0.67 ± 0.16*∗∗*	0.04 ± 0.01	1.05 ± 0.53*∗*

All values are means ± SEM.

**∗** *P* < 0.05, *∗∗P* <0.01, vs *db/m* mice at the same age, unpaired* t-*test.

## Data Availability

The data used to support the findings of this study are included within the article.
